# Mechanisms of HIV-immunologic non-responses and research trends based on gut microbiota

**DOI:** 10.3389/fimmu.2024.1378431

**Published:** 2024-12-26

**Authors:** Xiangbin Sun, Zhanpeng Xie, Zhen Wu, Meiyang Song, Youxian Zhang, Zezhan Zhang, Xinxin Cui, Aodi Liu, Ke Li

**Affiliations:** ^1^ Medical School of Shihezi University, Shihezi, China; ^2^ Department of Preventive Medicine, Medical School of Shihezi University, Shihezi, China

**Keywords:** HIV/AIDS, INRs, ART, gut microbiota, traditional Chinese medicine (TCM)

## Abstract

With the increasing number of people with HIV (PWH) and the use of antiretroviral treatment (ART) for PWH, HIV has gradually become a chronic infectious disease. However, some infected individuals develop issues with immunologic non-responses (INRs) after receiving ART, which can lead to secondary infections and seriously affect the life expectancy and quality of life of PWH. Disruption of the gut microbiota is an important factor in immune activation and inflammation in HIV/AIDS, thus stabilizing the gut microbiota to reduce immune activation and inflammation and promoting immune reconstitution may become a direction for the treatment of HIV/AIDS. This paper, based on extensive literature review, summarizes the definition, mechanisms, and solutions for INRs, starting from the perspective of gut microbiota.

## 1 Introduction

Acquired immunodeficiency syndrome (AIDS) is an infectious disease with immune deficiency caused by infection with HIV (1, 2). CD4^+^T cells are the target cells of HIV, the intestinal tract is a reservoir of HIV, Without using ART, resulting in low immune function of patients and subsequent death of various diseases (3, 4), for example, tumors, opportunistic infections. Recently, in ART used for PWH, HIV replication is suppressed, even HIV load below the detection level, but still about 15% -30% of patients in INRs (5), seriously affect the quality of life and life expectancy, INRs patients have higher non-AIDS events, such as the occurrence of malignant tumor (6, 7). With the development of research on INRs, more and more mechanisms of INRs have been discovered, treatments for INRs have been proposed, and better therapeutic results have been achieved in recent years. Therefore, this paper aims to provide direction for future solutions of INRs in PWH.

## 2 Definition of the INRs

Since the introduction of ART for PWH, the condition of PWH has been effectively controlled, resulting in improved quality of life and increased life expectancy (8). Additionally, there has been a significant reduction in viral load and a restoration or near normalization of CD_4_^+^ T cell counts, approaching levels seen in healthy individuals. However, there is still a subset of individuals in whom the CD_4_^+^ T cell count fails to increase or increases at a slow pace. This phenomenon is referred to as INRs (9, 10).

Currently, it is generally recognized that individuals who have been receiving ART for one year and have achieved undetectable viral RNA copies in plasma, but still have a CD_4_^+^ T cell count below 200 cells/ul, or have a CD_4_^+^ T cell count that has not increased by at least 20% from baseline, are classified as INRs. Additionally, there are individuals who, after receiving ART for 4-7 years, still have a CD_4_^+^ T cell count below 350 cells/ul, also considered as INRs (11, 12). However, the precise definition of INRs remains unclear.

## 3 The mechanism of the INRs

### 3.1 Time of infection and timing of treatment

Numerous studies have indicated that compared to younger patients, elderly patients are more likely to experience INRs (13). Research by Kiros et al. (14) demonstrated that the probability of INRs occurring in patients aged 50 and above is 1.97 times higher. This may be due to the thymic atrophy and function decline in elderly individuals, resulting in reduced immune function and diminished production of CD_4_^+^ T cells (15). Moreover, individuals with larger thymic volume are more likely to experience a recovery in CD_4_^+^ T cell counts, contributing to a lower likelihood of INRs. Additionally, the timing of initiating ART also impacts the occurrence of INRs. Thus, early initiation of ART is beneficial in reducing the incidence of INRs.

Zhang et al. (16) conducted a large-scale retrospective cohort study, which indicated that early initiation of ART is more advantageous in reducing the occurrence of INRs in PWH. Furthermore, the World Health Organization (WHO) recommended in 2016 that once diagnosed with HIV/AIDS, immediate initiation of ART is essential (17). The rationale behind this recommendation is that early initiation of ART is advantageous in reducing inflammatory responses and disturbances in gut microbiota, thereby lowering CD_4_^+^ T cell depletion and promoting immune restoration. Therefore, immediate treatment upon diagnosis of HIV/AIDS is crucial to prevent the occurrence of INRs.

### 3.2 Translocation of the gut microbiota

In individuals infected with the HIV, there is a significant increase in apoptosis of T cells in the intestines, leading to damage to the intestinal mucosal barrier and increased permeability. This imbalance ultimately results in dysbiosis of the gut microbiota and abnormalities in metabolite production, potentially causing increased intestinal permeability, chronic inflammation, and overactivation of the immune system (18). Studies indicate that following HIV infection, there is a shift in the predominant bacterial species in the gut from *Bacteroidetes* to *Prevotella* (19), influenced more by sexual behavior patterns (such as men who have sex with men) (MSM)rather than HIV infection itself (20). Additionally, HIV leads to decreased microbial diversity, enrichment of *γ-β-Proteobacteria*, and reduction in members of the *Clostridia* genus (21).

**Figure 1:**
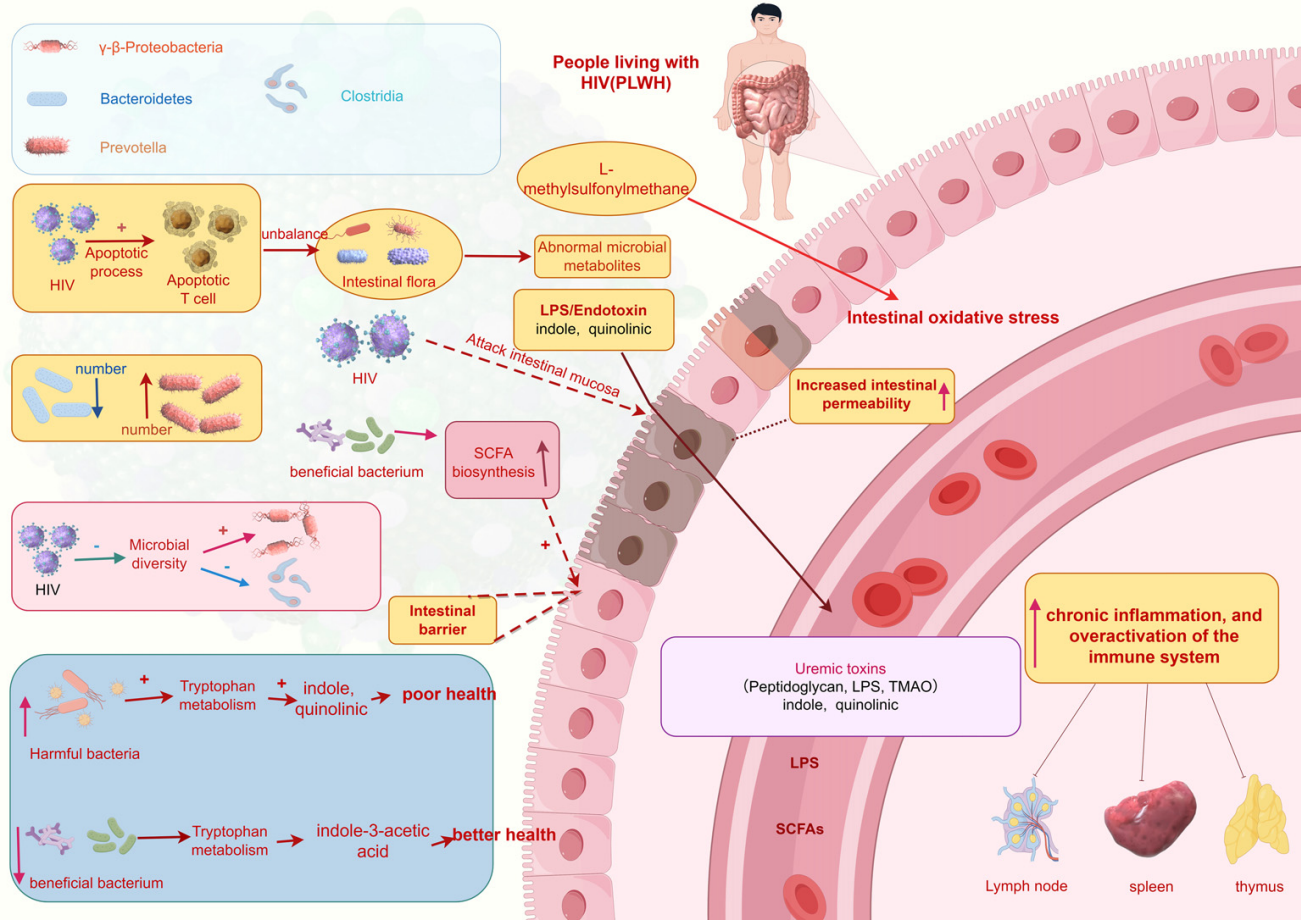
Translocation of the gut microbiota. (This figure is made by Figdraw.) (The patterns in the figure are only for effective differentiation and do not represent the actual appearance of the things referred to).

Overall, the gut microbiota in HIV-infected individuals shows a reduction in beneficial bacteria and an increase in harmful bacteria. Furthermore, HIV attacks the intestinal mucosa, further compromising the intestinal barrier and resulting in dysregulated microbial distribution, exacerbating inflammation and immune activation. Harmful bacteria such as *Catenibacterium*, *Prevotellaceae*, and *Enterobacteriaceae* can metabolize tryptophan, with metabolites such as indole and quinolinic acid associated with adverse health outcomes (22). Conversely, beneficial bacteria enhance intestinal barrier function by producing short-chain fatty acids, thereby reducing intestinal permeability and inflammation levels. Additionally, L-methylsulfonylmethane helps to reduce oxidative stress in the intestines (22). Beneficial bacteria also modulate host immune responses by affecting tryptophan metabolism rate and availability, producing beneficial metabolites like indole-3-acetic acid, which protects the host from the negative effects of excessive tryptophan on health (23) (**Figure 1**).

Heterotopic gut microbiota is an important factor affecting the degree of immune recovery after ART. Prevotellaceae is enriched in INRs, and its abundance is positively correlated with the activation of mucosal T cells (24). In addition, Ruminococcus is significantly reduced in INR, positively correlated with CD4^+^ T cell counts, and negatively correlated with serum pro-inflammatory cytokine levels (25). In addition, gut microbiota is also involved in the differentiation of T cell subsets (Th17 and higher regulatory T cells (Tregs)), and INRs has higher regulatory Tregs and lower Th17 percentage compared to immunological responders (IRs). The Th17/Treg ratio is negatively correlated with the levels of intestinal fatty acid binding proteins. Due to the continuous entry of bacterial lipopolysaccharides from the intestine into the bloodstream, immune activation and inflammation are exacerbated, leading to poor immune reconstitution.

### 3.3 Hyperactivation of the immune system

The disordered gut microbiota is characterized by an increase in pro-inflammatory bacterial species and a decrease in anti-inflammatory bacterial species (25, 26). Furthermore, bacterial lipopolysaccharides and inflammatory factors can both promote excessive immune system activation, virus replication, and the progression of HIV/AIDS, leading to damage to CD_4_^+^ T cells and the onset of non-AIDS defining illnesses (NADIs), thereby accelerating the course of HIV/AIDS. Excessive immune system activation is closely associated with mortality in HIV infection, and systemic chronic immune activation is considered to be a driving force behind CD_4_^+^ T cell depletion (27). Therefore, inhibiting excessive immune system activation may represent a new approach to addressing NADIs.

### 3.4 Platelet-T cell complexes

Dai et al. utilized flow cytometry analysis and immunofluorescence microscopy to observe that during HIV-1 infection, platelet-CD_4_^+^ T cell aggregates increase in treatment-naïve HIV-1-infected individuals (TNs) and INRs compared to healthy controls. However, the aggregation of platelet-CD_4_^+^ T cells in the INRs group did not decrease significantly compared to TNs and was associated with severe immune dysfunction. Platelet-CD_4_^+^ T cell aggregation positively correlated with HIV-1 viral load and negatively correlated with CD_4_^+^ T cell count and CD_4_^+^ T cell/CD_8_^+^ T cell ratio. CD45RO, HIV co-receptors, high levels of caspase-1 and caspase-3, and low levels of anti-apoptotic protein Bcl-2 were highly expressed in platelet-CD_4_^+^ T cell aggregates, which may contribute to CD_4_^+^ T cell depletion and sustained chronic inflammation in INRs (28). Zhu et al. recently demonstrated that platelets containing HIV in INRs can induce metabolic changes in CD_4_^+^ T cells through non-infectious mechanisms, specifically by forming platelet-CD_4_^+^ T cell aggregates (29–31). Enhanced glycolysis in immune cells and its contribution to ATP production are directly correlated with poor immune reconstitution. This effect specifically occurs in CD_4_^+^ T cells interacting with INRs platelets containing HIV. However, the mechanism behind this correlation may be due to INRs having a greater capacity for platelets to form aggregates with CD_4_^+^ T cells compared to IRs (28). Increased glycolysis is a crucial hallmark of T cell activation (32). Increased energy metabolism induced by platelets on T cells via INRs may contribute to the hyperactivation of CD_4_^+^ T cells, leading to CD_4_^+^ T cell exhaustion and triggering immune senescence associated with immune failure. Several mechanisms have been proposed to explain the immune modulation caused by platelet interaction with CD_4_^+^ T cells. Gerdes et al. suggest that platelet interactions with lymphocytes inhibit T cell proliferation and drive initial or memory CD_4_^+^ T cells towards regulatory (Treg: FoxP3^+^) or inflammatory (e.g., Th17) differentiation, thereby leading to immune failure (33). Platelets can induce polarization and/or secretion of chemotactic factors in lymphocytes through direct contact and interaction (34). Platelets can also release extracellular vesicles (microparticles) that directly interact with these lymphocytes, as well as myeloid cells and epithelial cells (35, 36). Functionally, these platelet-derived microvesicles can transfer active mRNA and microRNA (miRNA) to target cells, promoting the differentiation of T_reg_ (34, 37) (**Supplementary Figure S1**).

### 3.5 Co-infection

Negash, Hadush et al. (38) have shown that the rate of immune reconstitution failure in HIV-infected individuals co-infected with tuberculosis is significantly higher compared to those without co-infection. In addition to *Mycobacterium tuberculosis*, studies have also indicated that co-infection with hepatitis viruses is a contributing factor to NADIs in PWH (39). The mechanisms through which co-infections result in NADIs involve inflammatory cytokines, which trigger excessive immune system activation. Moreover, elevated plasma lipopolysaccharides lead to dysbiosis and translocation of gut microbiota, reducing the quantity and diversity of anti-inflammatory bacteria, which compromises the integrity of the intestinal mucosa. As a result, this reduction in intestinal T cell populations contributes to the development of NADIs.

### 3.6 Adipose factor

In obese PWH, inflammation, fat accumulation, and dysfunction interact with each other. In recent years, adipose tissue has been found to play a crucial role in regulating immune function in the body (40, 41). In fact, adipose tissue is considered a potential reservoir for HIV, and the persistent presence of the virus in adipose tissue may be related to metabolic and immune dysfunction in adipose tissue cells (42, 43). Elena Yeregui et al. conducted a multicenter prospective study evaluating the association of adipose factors such as apelin, apelin receptor (APLNR), and zinc-alpha-2-glycoprotein (ZAG) with poor immune recovery in PWH undergoing ART. The study found that concentrations of APLNR and ZAG were significantly lower in immunological INRs compared to responders, and these lower levels persisted during the treatment follow-up period. Levels of ZAG were positively correlated with levels of retinol-binding protein 4 (RBP4), and low circulating RBP4 concentrations were associated with poor CD_4_^+^ T cell recovery (44). ZAG is a novel adipokine primarily expressed in visceral and subcutaneous adipose tissue (45). Its role in the immune system may be mediated through its anti-inflammatory effects on T cells and macrophages (46). In addition, pro-inflammatory adipokines like leptin and resistin released into circulation may suppress the expression of anti-inflammatory ZAG protein through activation of inflammatory pathways such as TNF signaling. Therefore, the imbalance between circulating anti-inflammatory and pro-inflammatory adipokines in untreated and treated PWH may be crucial for the reconstruction of CD_4_^+^ T cell counts and subsequent immune responses. Apelin is the endogenous ligand for the apelin receptor (APLNR), which is a protein receptor secreted by adipocytes. Interestingly, apelin inhibits HIV entry into human cells by binding to the co-receptor APLNR in both T-tropic and HIV-1 strains (44). In this context, both apelin and APLNR are positively correlated with CD_4_^+^ T cell counts, supporting their association with HIV replication. Therefore, lower circulating concentrations of apelin and apelin receptor in PWH with immune failure may suggest a lack of inhibition of HIV entry into cells, thereby indirectly linking immune failure to the “reservoir” of persistent HIV in adipose tissue (47, 48).

### 3.7 Host metabolic levels

Although viral load decreases to a certain extent after ART, incomplete immune reconstitution and the associated chronic non-AIDS-related diseases remain a focal point in the treatment and recovery of PWH. Changes in host metabolic levels accompany HIV infection, raising questions about whether they can return to normal after ART and their relationship with immune recovery, which is a critical research area in immunoreconstitution. Previous studies have observed high accumulation of plasma acylcarnitines in INRs (49), and a persistent decline in the activity of sphingosine-1-phosphate phosphatase 1 (50). Lu et al. conducted a metabolomics and machine learning analysis comparing the metabolic profiles of healthy controls and PWH, both before and after long-term ART. They found that disruptions in lipid and nucleotide metabolism observed during HIV infection did not return to normal levels post-treatment. Only three metabolites—maltose, N,N-dimethyl-5-aminovalerate, and decadienoic acid—showed significant differences between IRs and INRs. Additionally, Qian S et al. discovered significant increases in medium-chain acylcarnitine, palmitoylcarnitine, stearoylcarnitine, and oleoylcarnitine levels in INRs (49). Another study found that high-density lipoprotein cholesterol and larger-sized low-density lipoprotein particles contribute to better immune recovery post-treatment (51). However, the sample sizes of identified metabolites in both studies were less than 20, and their conclusions were inconsistent, lacking generalizability. Additionally, the pathogenic reasons remain unclear. Therefore, the potential biological mechanisms of the metabolites described in the above studies in immune rebuilding still require further robust validation.

### 3.8 Other factors

Ge, Y et al. (52) conducted a retrospective cohort study and found that the mode of sexual transmission can influence the immune reconstitution after ART. It was observed that heterosexual men are more likely to experience NADIs compared to men who have sex with men. The bone marrow and thymus are vital for T cell production, and their impaired functionality can result in impaired T cell output, thereby affecting immune reconstitution (12).

## 4 The solution to the INRs

### 4.1 Gut microbiota intervention

As mentioned earlier, obesity plays a significant role in inflammation and immune regulation. The use of ART has aligned the obesity rates among PWH with those of the general population (53). Additionally, research by Gogokhia et al. indicates that regardless of the ART regimen used, the incidence of obesity continues to rise (54). However, different ART drugs have varying impacts on weight gain due to their effects on the gut microbiota (55). Therefore, investigating the influence of different drugs on the gut microbiota is crucial.

Imahashi et al. demonstrated that NRTIs significantly increase the β-diversity of the gut microbiota while reducing its α-diversity in PWH. Long-term use of NRTIs also leads to an enrichment of *Prevotella* species bacteria and a reduction in *Bacteroides* species bacteria (56). *Prevotella* species are known as pro-inflammatory bacteria and currently dominate the gut microbiota of PWH. The increase in *Prevotella* abundance raises the pH of the gut, creating a more favorable environment for HIV infection and replication, thereby enhancing the potential for bacterial colonization and transmission amplification (57). On the other hand, *Bacteroides* species are typical anti-inflammatory bacteria. Additionally, PWH treated with NNRTIs or INSTIs also exhibit reduced α-diversity and increased β-diversity of their gut microbiota (58, 59).

Interestingly, current studies on the effects of Protease Inhibitors (PIs) on gut microbiota yield inconsistent results. Some suggest that compared to NNRTIs or INSTIs, PIs have minimal impact on gut microbiota (60), while others indicate that PI treatment significantly affects microbiota, correlating with higher plasma levels of soluble CD14 and I-FABP (61). These differences may be influenced by confounding factors such as diet, antibiotic use, lifestyle habits, and ethnicity affecting gut microbiota. Moreover, as mentioned earlier, the predominant gut microbiota shifts from *Bacteroides* to *Prevotella* in adult infection cases linked to sexual behavior (MSM), whereas in children with HIV infection, *Prevotella* enrichment is a microbiological feature (62). Therefore, future studies need to correct for these confounding factors to determine how microbiota influences HIV capability and transmission.

**Figure 2:**
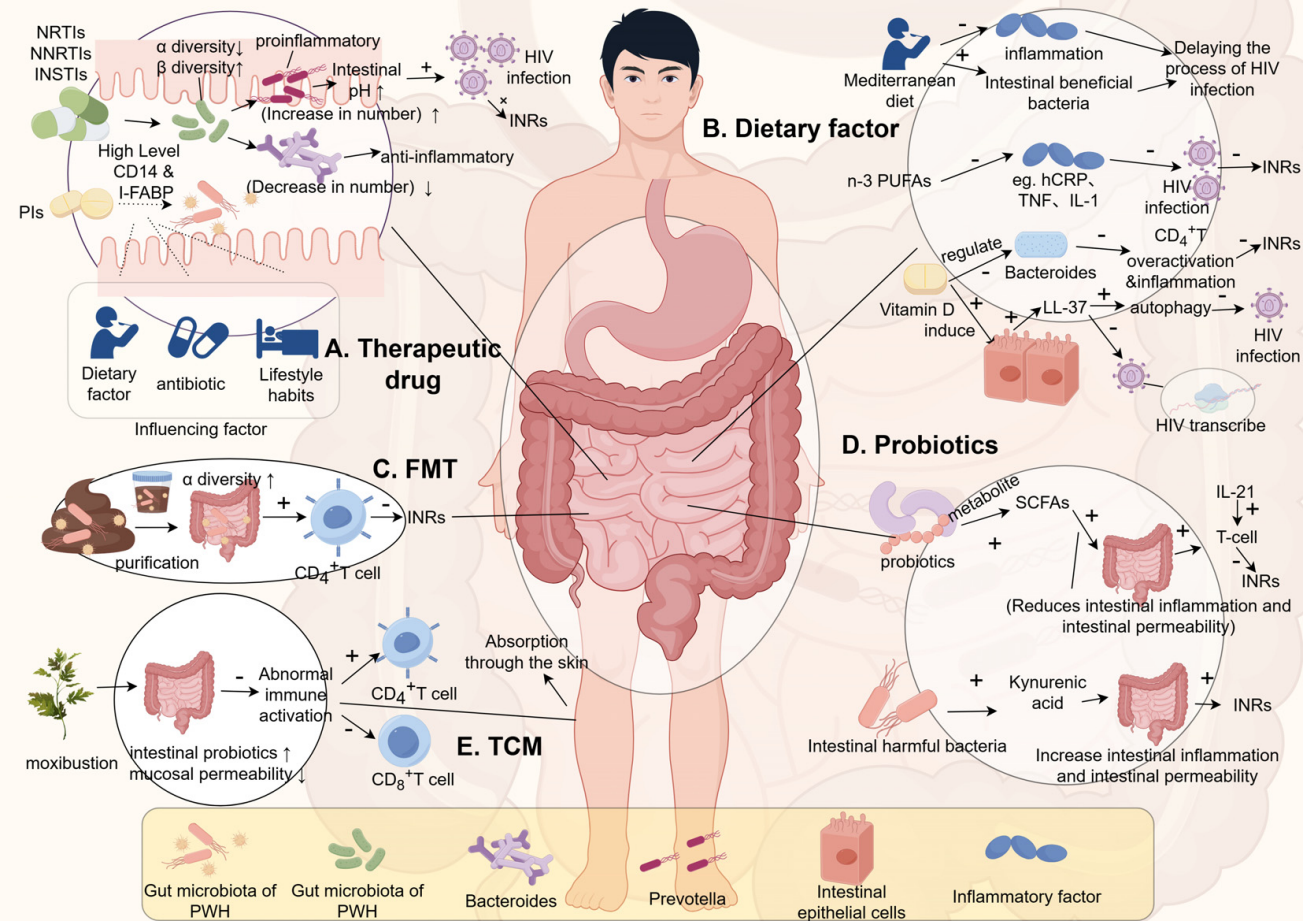
The relationship between gut microbiota and INRs. (This figure is made by Figdraw.) (The patterns in the figure are only for effective differentiation and do not represent the actual appearance of the things referred to). INRs, Immunologic non-responses; n-3 PUFAs, n-3 polyunsaturated fatty acids; FMT, fecal microbiota transplantation; SCFAs, short-chain fatty acids; TCM, Traditional Chinese Medicine; PWH, people living with HIV.

In conclusion, ART significantly reduces HIV viral load and restores CD_4_^+^ T cell counts, but immune function recovery remains limited in some patients. Thus, addressing immune restoration strategies from the perspective of gut microbiota is crucial for optimizing ART (**Figure 2A**).

The gut is a target organ for HIV infection as it contains a large number of CD_4_^+^ T cells, making it a reservoir for the virus. HIV alters the abundance and composition of the gut microbiota, characterized by a reduction in beneficial bacteria, such as *Bifidobacteria*, and an increase in pathogenic bacteria. The gut microbiota plays a crucial role in maintaining the integrity of the gut mucosa and the functionality of the gut mucosal barrier, which is closely associated with the development of Th17 helper T cells. Accordingly, improving the gut microbiota by increasing the abundance of anti-inflammatory bacterial species and reducing the quantity of pro-inflammatory bacteria may enhance CD_4_^+^ T cell count and improve immune reconstitution.

In a clinical randomized controlled trial, d’Ettorre et al. (63) administered probiotics to HIV-infected patients twice daily for 48 weeks, resulting in a decrease in T lymphocyte immune activation and inflammatory markers. Additionally, in a randomized double-blind study by Mortezazadeh et al. (64), the consumption of probiotic yogurt was found to increase T cell levels. However, the impact of probiotic supplementation on T cell count remains inconsistent in different meta-analyses, with some studies suggesting a positive effect while others demonstrating contradictory results (65, 66).

Although the supplementation of probiotics or prebiotics alone cannot reduce HIV viral load, they can contribute to the reduction of immune system overactivation and inflammation caused by gut permeability. Therefore, supplementing with probiotics or prebiotics is beneficial for restoring gut barrier function (67), thereby slowing down the progression of HIV, reducing the occurrence of NADIs, increasing patient survival expectancy, and improving overall quality of life (68–73).

Some studies have indicated that the effects on T cells vary between the use of individual probiotics and combination therapies. Concurrent use of probiotics and IL-21 during probiotic supplementation has been shown to be more favorable for immune reconstitution. Hence, future research may consider exploring the ratio of relevant probiotics or prebiotics and their combination, as well as the effects of different types of probiotics and interleukins on immune reconstitution (**Figure 2D**).

It is important to note that different probiotics have varying effects, and the aforementioned meta-analyses did not classify the types of probiotics used, which may introduce bias. Moreover, current research standards for probiotics are not yet well-defined, such as intervention durations, which have varied from weeks to years across different studies. Thus, future research should establish clear guidelines regarding probiotic types, intervention durations, and other relevant criteria.

Some studies have suggested that metabolites produced by the gut microbiota can have different effects on the host’s immune system. Short-chain fatty acids (such as butyrate) produced by probiotics can help maintain gut mucosal barrier integrity, decrease inflammatory responses, and reduce immune system activation. This, in turn, can help prevent the loss of CD_4_^+^ T cells and contribute to immune reconstitution (74). On the other hand, uric acid produced by the gut microbiota can disrupt the gut mucosa, impair gut immunity, and contribute to INRs. Therefore, increasing the levels of short-chain fatty acids in the body, as an adjunct to ART therapy, may potentially reduce the occurrence of INRs (**Figure 2D**).

Currently, sequencing techniques for the gut microbiota mainly rely on genomics and metabolomics, with limited use of proteomics. Future research may require the integration of multiple “omics” approaches to determine the roles of different metabolites in HIV immune reconstitution. This comprehensive approach could provide new insights and potential solutions for addressing INRs.

In recent years, fecal microbiota transplantation (FMT) has shown promising results in improving the gut microbiota. FMT has been found to significantly increase the alpha diversity of the gut microbiota, which is decreased in HIV-infected individuals (75). Furthermore, individuals with lower CD4+ T cell counts also exhibit reduced alpha diversity of their gut microbiota, indicating a significant correlation between gut microbiota diversity and immune status. Moreover, studies have demonstrated that FMT can significantly increase CD4+ T cell counts (76), suggesting that transplanting probiotics into the gut of HIV-infected individuals may reduce the occurrence of INRs. Plasma levels of inflammation-associated proteins in INRs were significantly higher than those in IRs. Moreover, these biomarkers were negatively correlated with the CD4+ cell count and positively correlated with the HIV viral load (77). A recent authoritative omics study published by Diaz-Garcia, C. et al. demonstrated that FMT could change the gut microbiota by targeting (the *Ruminococcaceae*, *Succinivibrionaceae*, *Prevotellaceae* families, and the *Clostridium* genus, etc.) to effectively reduce the levels of 46 inflammatory proteins such as IL6 in PWH (78). Repeated oral FMT significantly enhanced the levels of intestinal fatty acid-binding protein (IFABP), which has been demonstrated to be an independent predictor of mortality as a biomarker of intestinal damage (79). Therefore, the more stable intestinal barrier and the lower inflammation level formed by FMT might further decrease the level of INRs in PWH or reduce the early occurrence of INRs. With a stricter and more standardized study design and a more innovative proteomic assay, this research constitutes an important step in the exploration of longitudinal associations between fecal bacteria and plasma-associated proteins. However, considering the complex immune system regulation and the unclear influence mechanism of FMT on PWH, we should further clarify the regulatory mechanism and functional level of the targeted gut microbiome in PWH inflammation through transcriptomics and metabolomics in the future. However, there are still many selective and controversial issues that need to be addressed regarding FMT. For example, it is essential to investigate whether the newly formed microbiota after transplantation can be maintained in a stable state for the long term, rather than just in the short term. Additionally, it is important to determine if the transplanted microbiota is highly compatible with the host, rather than exacerbating immune reactions and stimulating immune system activation. Factors influencing the stability of the transplanted microbiota also need to be studied. Therefore, large-scale longitudinal studies are needed in the future to explore the role of FMT in immune reconstitution in HIV-infected individuals. However, neither FMT nor the intervention of probiotics and prebiotics has provided clear evidence for reducing chronic inflammation and immune system activation in HIV. Further research is still required to explore these areas. Additionally, the gut microbiota consists not only of bacteria and fungi but also of viral communities. Compared with non-pathogenic SIV, enteric virome expansion has been found in pathogenic SIV, and plays a role in immune deficiency (80). Transplanting the gut viral community from healthy individuals has shown benefits in treating recurrent Clostridium difficile infection, alleviating diet-induced obesity, and preventing necrotizing enterocolitis in premature infants (81). Therefore, whether immune deficiency is involved in PWH by enteric virome expansion. However, there are currently no reports on the transplantation of the viral community in PWH. Therefore, investigating the effects of viral community transplantation on the immune deficiencies of PWH should be explored in the future (**Figure 2C**).

### 4.2 Traditional Chinese medicine intervention

TCM can enhance patients’ immune function, providing a new potential solution for individuals with INRs (82). The gastrointestinal tract serves not only as an immune organ but also as a digestive organ. Following absorption in the gastrointestinal tract, TCM can impact the metabolism of the gut microbiota. Simultaneously, the gut microbiota influences the bioavailability of TCM, promoting the proliferation of beneficial bacteria while reducing the abundance of harmful bacteria (83, 84).

Mechanistic studies on TCM therapy for PWH primarily focus on alterations in the gut microbiota, damage to the intestinal mucosal barrier, and changes in CD_4_^+^ T lymphocytes (85, 86). TCM has the capacity to enhance patients’ immune function, reduce complications, and improve survival rates and quality of life (87).

The combined use of traditional Chinese medicine and ART for PWH can offer complementary advantages (88). Adopting an integrated approach to treatment involving both TCM and ART can provide valuable insights into immune reconstitution and viral reservoir clearance in PWH (89).

Moxibustion therapy mainly achieves its therapeutic effects through the warmth and stimulation produced by the burning of moxa, as well as the absorption of moxa particles and aroma by the skin or through inhalation. Moxibustion offers the advantage of multi-targeted and bidirectional immune regulation in the human body. It can correct abnormal immune responses in the intestines, increase the population of beneficial probiotics in the gut microbiota, and reduce the permeability of the intestinal mucosa through the metabolic byproducts of probiotics (90). Additionally, it can lower the occurrence of abnormal immune responses, maintain intestinal stability, and prevent excessive T-cell activation. Therefore, moxibustion has a direct or indirect impact on the intestines of PWH. Moxibustion can increase the CD_4_^+^ T cell count in patients, while also reducing the number of CD_8_^+^ T cells, restoring the normal ratio of CD_4_^+^ T cells to CD_8_^+^ T cells, enhancing immune function, reducing the production of inflammatory factors, and improving INRs. Furthermore, moxibustion is a non-invasive treatment that can effectively prevent healthcare-associated infections caused by bloodborne pathogens in the context of HIV (**Figure 2E**).

In recent years, research on the use of Artesunate for PWH has become increasingly frequent. Chen et al. (91) conducted a clinical randomized controlled trial, administering Artesunate orally to PWH. After 48 weeks, the study found a reduction in T-cell activation markers and a decrease in T-cell apoptosis levels. However, there was no improvement in T-cell count. On the other hand, Artesunate also improved the gut microbiota of infected individuals, increasing the abundance of probiotics such as *Actinobacteria* and *Bifidobacterium*. Moreover, a higher level of CD_4_^+^ T cells in peripheral blood was associated with an increased level of *Actinobacteria* in the gut microbiota. Additionally, Artesunate appeared to have no significant clinical effect on immune reconstitution in younger individuals, but had a more pronounced effect in middle-aged and older individuals, suggesting that Artesunate may improve thymic atrophy and therefore contribute to the improvement of INRs.

Liu et al. (92) administered Tripterygium wilfordii to PWH with immune non-response for 17 months, and observed an increase in CD_4_^+^ T cell count along with a decrease in T cell activation. Through multi-omics studies, the mechanism was found to involve the inhibition of the interferon signaling pathway. Genomics is currently widely used in research on the role of the gut microbiota in HIV/AIDS. However, genomics has focused excessively on microbiota differences, and genomics and transcriptomics are primarily used for functional predictions. Nevertheless, predicted functions do not necessarily translate into actual expression. Therefore, future research should incorporate transcriptomics and proteomics to specifically investigate their roles and underlying mechanisms (93).

### 4.3 Dietary intervention

Diet is also an important pathway for altering the gut microbiota. Manzano et al. (94) compared the differential effects of the Mediterranean diet and the Western diet for PWH treatment and found that the Mediterranean diet can reduce inflammation levels while preserving beneficial bacteria. Although no specific dietary pattern has been found to increase CD_4_^+^ T cell counts, dietary changes can lower inflammation levels and slow the progression of HIV/AIDS. Currently, there is a lack of specific dietary interventions for the treatment of PWH, and further research is needed to explore this area in the future.

#### 4.3.1 Vitamin D

In recent years, multiple studies have demonstrated that nutrient compounds such as vitamin D, which possess immune-regulatory properties, play a significant role in maintaining intestinal homeostasis through their effects on both innate and adaptive immunity (95, 96). Vitamin D can induce the antimicrobial peptide LL-37 from epithelial cells and immune cells (97). LL-37 exhibits multifaceted protective effects that can be enhanced by vitamin D (98). In the intestinal mucosal barrier, the production of LL-37 serves as an important natural defense mechanism primarily by activating autophagy to delay the progression of HIV disease. Autophagy reduces intracellular HIV replication (99). Alternatively, it can delay infection by inhibiting HIV-1 transcription (100, 101). Additionally, vitamin D can stabilize the tight junction structure of intestinal epithelial cells (102). And may also regulate the composition of human gut microbiota. Recent studies suggest that vitamin D may modulate the relative abundance of pro-inflammatory *Bacteroides* species in the gut (103, 104). Raftery et al. and Ponda et al. separately found that vitamin D treatment is associated with reduced systemic inflammation levels and disease activity in inflammatory bowel disease and chronic kidney disease (105). However, Missailidis et al., using randomized controlled trials, found that vitamin D + phenylbutyrate supplementation did not improve markers of intestinal inflammation or gut microbiota composition in treatment-naive individuals with active HIV-1 replication. This lack of effect may be partly attributed to ongoing viral replication in untreated HIV. Future studies should investigate the supplementation of vitamin D in individuals receiving ART for HIV infection more extensively, to better assess its regulatory role in a controlled viral environment (106) (**Figure 2B**).

#### 4.3.2 Reduction of the refined sugars and saturated fatty acids

The progression of HIV infection is often associated with various metabolic and cardiovascular complications (107). These complications may be related to the side effects of ART, but HIV infection itself, even during successful ART, can induce metabolic changes through mechanisms such as chronic low-grade systemic inflammation (108, 109). Fatty acids (FAs), whether free or as part of other lipids such as triglycerides, phospholipids, sphingolipids, and sterol lipids, play critical roles in cellular energy metabolism and are essential components of cell membranes. FAs also have the ability to signal through peroxisome proliferator-activated receptors (110, 111). Disruption in the composition of FAs has been recognized to impact the development of various metabolic, cardiovascular, and inflammatory diseases (112). For example, certain polyunsaturated fatty acids (PUFAs) such as arachidonic acid (AA; C20:4n-6), eicosapentaenoic acid (EPA; C20:5n-3), and docosahexaenoic acid (DHA; C22:6n-3) serve as precursors for the synthesis of biologically active lipid mediators (e.g., prostaglandins [PGs], leukotrienes [LTs], lipoxins, and resolvins) (113, 114), mediating both inflammatory and anti-inflammatory effects (114, 115). There is also significant focus on the central role of fatty acids in regulating immune cell function, emphasizing their direct impact on many cellular processes involved in T cell responses and antigen presentation (116–118). There is evidence suggesting that FA metabolism is disrupted in PWH. Additionally, a recent meta-analysis indicated that supplementing n-3 polyunsaturated fatty acids (n-3 PUFA) may alleviate inflammation in PWH, as assessed by levels of C-reactive protein in patients receiving ART (119). In an earlier meta-analysis, it was shown that supplementation with n-3 polyunsaturated fatty acids can lower triglyceride levels in PWH undergoing ART (120). Furthermore, several studies suggest that n-3 polyunsaturated fatty acids (n-3 PUFA) may inhibit classical inflammatory cytokines such as tumor necrosis factor (TNF), interleukin (IL)-1, and IL-6 (121–123), and are associated with HIV-related pathogenic mechanisms involving these cytokines (124). Therefore, PWH at any stage should consume foods rich in unsaturated fatty acids and minimize intake of refined sugars and saturated fats commonly found in Western diets (125) (**Figure 2B**).

## 5 Summary

In recent years, the number of PWH has been increasing year after year, and research in the area of INRs has gradually intensified. In this paper, we formulated the definition of INRs, expounded the mechanism underlying the association between gut microbiota translocation, platelet-CD_4_^+^ T cell coupling, and host metabolism with immune failure in INRs, and further explored potential therapeutic interventions for INRs gut microbiota. In the future, we will endeavor to provide a more distinct and comprehensive definition of INRs. However, at present, there is a dearth of large-scale data and evidence from evidence-based medical research to support approaches targeting INRs. Therefore, future studies should focus on the mechanism of the gut microbiota for immune reconstitution in INRs and make use of advanced technologies such as multi-omics analysis to conduct in-depth research in all aspects of this field.
