# Supplementary figures and images for: Mechanisms of HIV-immunologic non-responses and research trends based on gut microbiota
Source: Front Immunol. 2024 Dec 26;15:1378431. doi: 10.3389/fimmu.2024.1378431 (PMC11718445; doi:10.3389/fimmu.2024.1378431)

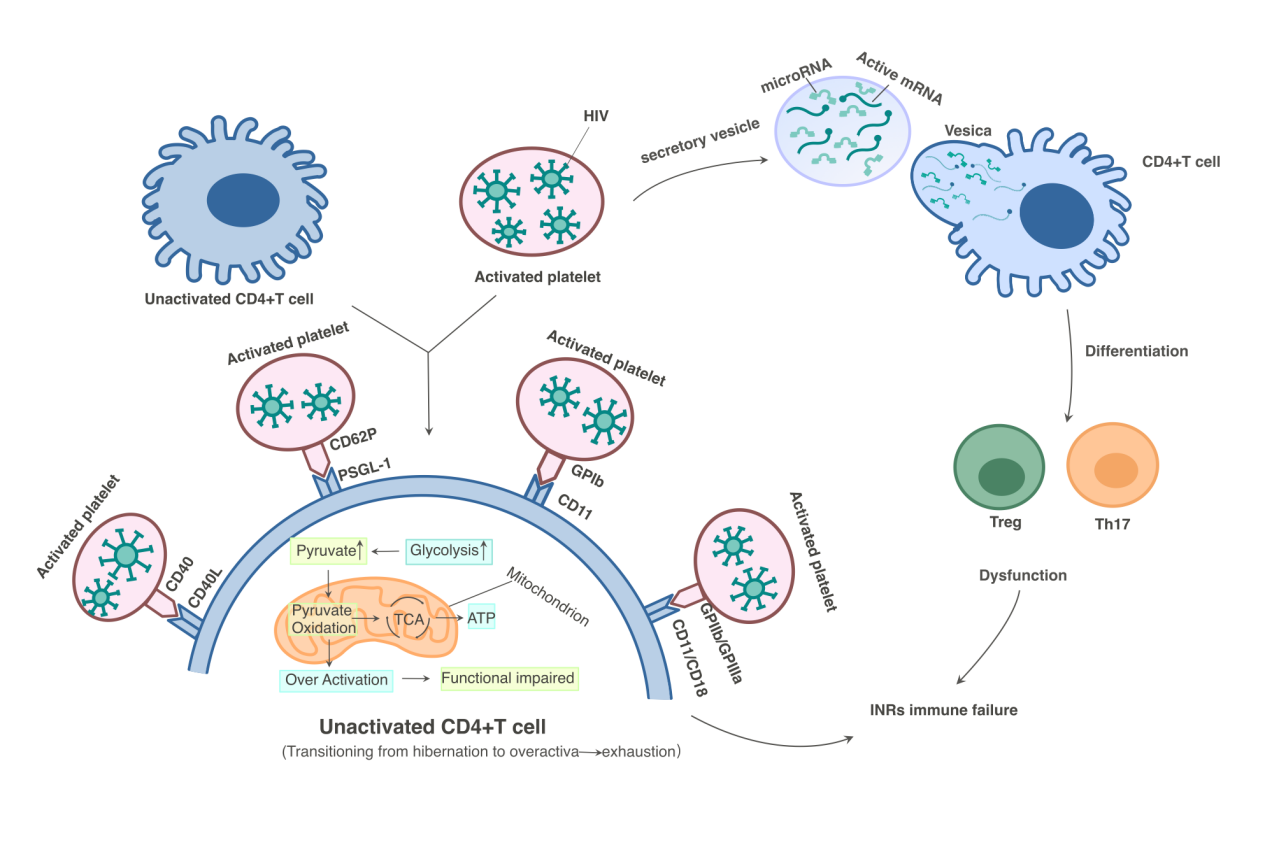


**Spplementary figure 1.** Mechanism of interaction between platelets and CD4+T cells

Supplement: Supplementary file 1 [file DataSheet1.docx]
